# Mr. Swan, Dr. Kerrison, and Mr. Villers, on Affections of the Nerves

**Published:** 1821-06-01

**Authors:** 


1. *A Dissertation on the Treatment of Morbid Local Affections of Nerves: to which the Jacksonian Prize was adjudged by the Royal College of Surgeons.* By JOSEPH SWAN, Member of the Royal College of Surgeons, and Surgeon to the Lincoln County Hospital. One vol. 8vo, pp. 196, three plates. London, 1820.

"Non Scribo hoc temere. Quo minus familiaris sum, hoc sum ad investigandum curiosior." *Cicero.*

2. *Tentamen Medicum inaugurale de Neuralgia faciali Spasmodica.* By ROBERT MASTERS KERRISON, M. D. Licentiate of the Royal College of Physicians. Octavo, sewed, pp. 45. Edinburgh, 1820.

"Cognitis indiciis, quæ nos vel spe consolentur, vel metu terriant, ad curationes morborum, transeundum est." *Cels.*

3. *A Treatise on that painful Disease of the Face, called Tic Douloureux: including Practical Observations and Illustrations tending to point out not only a Plan of Prevention, but of* radical *Cure; with Formulæ of Prescription used successfully.* By G. H. VILLERS, Physician-Accoucheur, formerly a Surgeon in the Army. Octavo, pp. 34. London, 1821.

THE sentiment of the great Roman orator, expressed in Mr. Swan's motto, ought especially to actuate the medical philosopher; for, in no province of human knowledge is there greater scope for investigation than in that of medicine and its auxiliary branches. The anatomy of the nervous system, it is true, has been cultivated with considerable success by many eminent men of former and present times; but a dense cloud overhangs its physiology, and has hitherto veiled most of its laws in almost total obscurity. Of all the neurological theories that have been proposed, Mr. Swan is disposed to consider that one the most rational which goes to the identity, or at least great similarity, of the nervous and electric fluids—a presumption, he thinks, greatly strengthened by the circumstance that the galvanic influence on the nerves of an animal apparently dead, will produce the same motions in the parts to which these nerves are distributed, that were produced in them when the animal was alive. Without questioning the considerable analogy which subsists between the nervous and galvanic fluids, we are disposed to view these experiments with some degree of scepticism. A needle pricking the muscle of a dead animal, will often cause it to contract, and we consider the galvanic shocks thrown along the nerves of a dead animal, as only so many powerful stimuli, but not by any means identifying them with the fluid, or whatever it may be which is transmitted from the brain to the muscle in the living body. The fact of the torpedo and gymnotus electricus being able to communicate shocks to other animals is much more presumptive proof than the galvanic experiments; yet even here we are hardly authorized to infer similarity of cause from similarity of effect.

The strongest proof of the analogy in question has undoubtedly been drawn from the action of secretion being renewed and carried on by aid of galvanic influence, after the nerves going to the secretory organs have been divided. But these physiological disquisitions we shall leave to the many able experimenters who are now directing their investigations towards that important point.

The sanction of the Royal College of Surgeons, as evinced by the Jacksonian prize, is in itself a passport, for the work before us, to public patronage; but we believe that the analytical sketch which we shall forthwith present our readers, will not only accelerate the circulation of the work itself, but diffuse a knowledge of its valuable contents to an extent which it would not otherwise be likely to reach.

The work is divided into fifteen chapters, the contents of which we shall not enumerate here, as we shall dip, more or less deeply, into each as we pass along.

I. The first chapter is dedicated to diseases and injuries of the nerves of sense. These, our author remarks, do not seem to suffer much from injuries and diseases—or at least they do not excite so much constitutional disturbance as affections in other parts of the nervous system—yet, when once affected, they are less disposed to the restorative process than other nerves. The olfactory functions may be diminished or destroyed by the frequent application of strong odours, or by inflammation of the schneiderian membrane, or pressure on the origins of the nerves by hydatids, &c. In the case of inflammation, leeches to the exterior of the nose, cooling ointment to the interior of it, and purging medicines, are the proper means. A case is related of a man who complained of very violent pain in the forehead for many days, especially towards the right side of the crista galli, with complete loss of smell in the right nostril, that of the left remaining entire. He was bled copiously from the arm and temples, took antimonial powder with submuriate of mercury and sulphate of magnesia, besides being put on a strict regimen. By these means the pain gradually declined, and the sense of smell returned. The functions of the olfactory, as of other nerves of sense, are sometimes so disordered as to produce unpleasant sensations, and that without any manifest cause, excepting that the functions of the stomach and associated organs are generally deranged. These functions restored, the sympathetic disorder generally ceases.

We shall pass over Mr. Swan's observations on affections of the optic nerves constituting amaurosis, since these are more fully treated of in our analysis of Mr. Travers's excellent volume.

In the following case our author had reason to suppose that there was a fracture of the base of the skull, that the petrous portion of the left temporal bone was much injured, and that the portio mollis of the seventh pair was destroyed. As the case is short, and rather interesting, we shall quote it.

"A man fell from a loaded waggon, and pitched on his head on the left parietal bone. A small wound was made, but this bone was not injured. Much blood flowed from the left ear, and a little from the right, and he became insensible. On being bled he became more sensible, but the next day he was again insensible. His pulse was eighty, and weak. Four grains of submuriate of mercury and purging medicines were given him. The third day he kept sleeping, but when roused appeared more sensible. The purging medicines had operated, and his pulse was seventy-two, and weak. Four grains of the antimonial powder and a saline draught were given every four hours. On the fifth day he became quite sensible, but had pain in his head and was entirely deaf. On the seventh day the pain in his head had increased, his pulse was only fifty-four, and his cheeks were flushed. Six ounces of blood were taken from the arm, which relieved him. On the eighth day he continued better, though his pulse was only fifty-four. On the tenth day he kept mending, but when he attempted to walk, his legs appeared very weak. He continued entirely deaf. A great quantity of an aqueous fluid had kept constantly discharging from his left ear, and some from his right.

"On the second day after the accident the fluid was very much tinged with blood, but after that it kept gradually getting paler. On the tenth day it was quite pale. I collected some in a tea-spoon, and made it boil over a candle, but it did not coagulate, and it was saltish to the taste.

"Some months after when I saw him he could hear tolerably with the right ear, but remained perfectly deaf of the left." P. 12.

We must pass over a number of cases and observations respecting disorders of the auditory nerves, from page 12 to 29 of the work, part of which have been read in or published by the Medico-Chirurgical Society.

II. In the second chapter our author takes up diseases and injuries of the nerves of voluntary motion in general.

It is this class which principally suffers in paralytic affections, though the nerves of sense in the skin arise with the others going to the muscles. Mr. S. endeavours to account for this phenomenon "by supposing that the muscles of voluntary motion require the nerves to be in the most perfect state to enable them to act, and that a less degree of perfection is necessary for them to perform the functions required for the sense of feeling."

"When much pressure," says he, "is made on the medulla spinalis, as in most fractures of the spine, all the nerves below the injury lose entirely the power of communicating either sensation or motion to the parts to which they are distributed. When the pressure has been rather less, and some power of transmitting the nervous influence is left, it is sensation in different degrees that is produced. When the pressure is still less, there is along with sensation a feeble power over the muscles; and these circumstances, I think, go to prove what I have stated already, viz. that when the functions of the nerves of a limb are impaired, as in paralysis, they must all equally suffer; and the apparent difference of the effects of the paralysis in the several parts affected by it, arises from the different degrees of perfection necessary for enabling each of them to perform their functions." 31.

It sometimes is the case, but comparatively rare, that the nerves of sensation suffer from paralysis, while those of voluntary motion, though arising from the same trunks, are little affected. When this occurs, our author thinks that such an alteration takes place in the skin as prevents the proper exercise of the nerves distributed to it. We know, indeed, that the sense of feeling will vary according to the state of the skin, and particularly of the blood-vessels.

"In further support of the opinion, I might mention the different constructions of the organs or parts themselves necessary for furnishing the nerves with a proper supply of blood; but I will merely describe the curious structure in the nose, which appears to me to be formed for the perfection of the sense of smell.

"Beneath the Schneiderian membrane there are numerous sinuses, and many of them of considerable size, which have frequent communications with each other, and appear to be composed of a very thin and inelastic membrane, which is very strong, and perfectly smooth in the inside: within the sinuses are contained very delicate and extremely elastic vessels, which may be called veins, as they appear to be filled with venous blood; and by their being thus situated within sinuses of a determinate size, they are capable of being distended to a certain degree only; which provision is necessary, as their extreme delicacy would otherwise either endanger their very frequent breaking from over-distension, or be the cause of much injury to the very delicate nerves by a too great pressure that would be thus made on them.

"This structure, I have no doubt, generally exists in animals, and may be very satisfactorily demonstrated in the horse; and it must, I think, appear to any one examining its peculiarities attentively, that it was not formed merely for returning the blood from the nose, but that it was made for distending the Schneiderian membrane, so as to give it a proper degree of tension to enable the nerves to receive more acutely the impressions from the odorous particles when applied to them; exactly in the same manner that it is required for the nerves of the penis to produce their peculiar sensations, that the parts connected with them should be properly distended with blood." 34.

III. Passing over physiology, we come to the pathology of the nerves of voluntary motion, in the 3d chapter of the work before us. These diseases are of two kinds, active and passive, or painful and paralytic. In the *former* class there is an increased action of the blood-vessels, and also of the animal temperature of the apparent seat of disease—in the *latter* class there is a contrary state. In the following sentiments we entirely agree with our intelligent author.

"Those local complaints which appear to originate spontaneously, or in some cases where a slight wound has been inflicted, I believe to be only symptomatic of a general irritability of the brain and nervous system. The almost constant failure of topical remedies, and of the division of the affected nerve, must lead to the conclusion that the cause of the local diseased action, or primary affection, must reside in some other part of the body: and if we inquire into the causes of the local active affections of the nerves, it will be found that the atonic state of the body, or whatever tends to render the brain and nervous system irritable, will generally be found the most frequent." 38.

This irritability of the brain, Mr. Swan observes, is produced by any excessive exertion of its powers, such as too great attention to business, the depressing passions, irregularity of living, inordinate use of fermented liquors, disordered states of the digestive organs, &c. The stomach being the centre of sympathies, there is no part of the body that is not liable to be affected by the state of this organ. "But why part of a nerve should suffer without any alteration in its organization is almost inexplicable." On this sentiment we would remark that we are by no means authorized to affirm that there is no actual, because no apparent, alteration of structure in a nerve; and moreover as the pain is generally referred to a place distant more or less from the seat of irritation or lesion, we cannot expect to find the alteration of structure at that part, if it at all exist.

IV. The fourth chapter is dedicated to those painful affections of the nerves about the head and face which have been variously denominated, as clavus hystericus, hemicrania, tic douloureux, &c. "appearing to be all the same disease, only varying in situation and degree." The symptomatology of this class of affections is unfortunately too well known. The pain, though sometimes attacking the whole, or great part of the head, is generally confined to one side only, and frequently to but a part of that, as one eye, the upper lip and nose, the gums, &c. varying in different people, from a common head-ache to the most exquisite anguish which human nature can possibly suffer without destroying life. "Volat enim angor per nervi surculos fulgure ociùs, desinitque sensu vibrante, ægrotum miserimum attonitumque subitò relinquente."*

"When the pain is confined principally to the head, and has become almost continual, it may be suspected that there is some disease within the cranium; on the contrary, if there is a perfect intermission in the twenty-four hours at about the same time, or if the pain, though constant, becomes more violent once in twenty-four hours at about the same hour, and continues so for some time, and then somewhat abates; if it strikes down the face, and wine does not increase it, and if the patient has been bled from the arm, and has experienced no relief; in all these cases, it may be judged to be an affection of the nerves without the cranium." 43.

Anatomy has done but little, as yet, in explaining the exact nature of tic douloureux; for, although it generally happens that this complaint, especially in its progress, is attended with an increase of heat, and also of vascularity in the part affected, yet it appears to Mr. Swan that these are the *effects* of the nervous irritation, which, however, may tend to increase and keep up their cause. The nerves may become enlarged from irritation, in the same way the muscles are from continued action; but dissection, our author observes, has not shown those depositions of coagulable lymph, and changes of structure produced by continued inflammations of other parts of the body, and of the nerves themselves in stumps and portions long the seat of inflammatory action. This is also the opinion of Dr. Kerrison, as expressed at page 6 of his able thesis.

"Sometimes it is produced by irritation, as from an ulcer connected with a branch of a nerve; sometimes from a decayed tooth, from the anastomosis between the affected nerve and those of the teeth, but most frequently from some disorder of the constitution." P. 45.

---

* Dr. Kerrison's Thesis, page 9

Tic douloureux appears to Mr. Swan a diametrically opposite affection to that of paralysis.

"We find that debility of the body is a state the most fitted for keeping up an irritation of the nervous system; and when any part of it has once become irritated in a subject to which we might suppose there was a predisposition, a habit is formed so as to continue the irritation, which generally becomes stronger and more obstinate, the longer it is unopposed by such remedies as have the power of breaking through it." 46.

Sometimes the complaint terminates spontaneously, sometimes by the supervention of another disease, of which Mr. Swan relates an instance at page 46 of his work. The patient was 43 years of age, and had received a blow on the right eye, which produced a great extravasation of blood between the conjunctiva and sclerotica. At first he complained of very violent pains in the eye, for which he was bled, purged, and had cooling washes. Shortly afterwards he complained of stabbing or darting pains, which went from the temple down the face, and sometimes to the ear, coming on in violent paroxysms, and always confined to the nerve. His complaint continued ten weeks, at first almost constant, then twice or thrice a day. At length an eruption, resembling the nettle rash, came all over his body, when the pains instantly ceased, and returned no more.

"During all the time before the eruption his spirits were much depressed. Much anxiety always increased the paroxysms, as did likewise any thing that caused him to be angry; much stooping, or motion of the head, likewise brought them on. During the paroxysms there was a great pulsation of the temporal arteries." 47.

There appears to Mr. Swan two principal indications of treatment in this disease—1st, to strengthen the constitution, and thereby enable it to counteract the habit which favours the continuance of the irritation—the 2d indication is to allay the local irritation.

"The first is best fulfilled by the exhibition of tonic remedies in doses, which must be repeated frequently, and at regular intervals, so as to produce new and regular actions: and when the diseased action is very violent, sedatives must be given, both with a view of alleviating the pain, and assisting the constitution to overcome the morbid actions. The best tonic remedy for affecting this change is bark, which should be taken regularly in doses, from half a drachm to a drachm every three or four hours, day and night: wine and malt liquor should be allowed rather freely. In this complaint the digestive organs are frequently disordered, but I have often known them restored during this plan of treatment." 48.

Mr. S. thinks arsenic a doubtful remedy, and that mercury should be used very sparingly. In addition to remedies the patient should take regular and gentle exercise, and endeavour to tranquillize the mind, an endeavour, by the bye, that is generally fruitless, for few can call in the precepts of philosophy, or even of religion, to their aid, when labouring under severe corporeal pain! The following case is related by our author to shew that tic douloureux and periodical face-ache are the same affection of the nerves; and also for the purpose of shewing the manner in which the bark should be administered to produce its beneficial effect.

"Mrs. P. forty-eight years of age, had complained of a violent pain in her face, which extended up the head, and was supposed to proceed from the stump of a tooth. On the eighth day after its first attack I saw her; she complained of very excruciating pain, which came on at nine every evening: it was not the continued pain of the tooth-ache, but came on in excruciating paroxysms, which lasted two or three minutes, and then abated. She would continue easy for a few minutes, and then again the pain would recur; and in this manner she passed her time until seven in the morning, when she became quite easy, though the pain would return occasionally at different times in the day. It seemed to be confined to the upper branches of the portio dura of the seventh pair of nerves. As it was supposed to proceed from a tooth, the tooth was extracted; and as her mouth was inflamed, she took four grains of submuriate of mercury and some purging medicine. On the tenth day I saw her again, when she had received no benefit from what I had done for her. I ordered her the following draught, to be taken half an hour before the pain came on, and the powders to be taken regularly every three hours through the day and night.

"℞. Tinct. Opii. gtt. xl.

Liq. Ant. Tart. gtt. xv.

Aq. Puræ. ℥j. Mf. Haust.

℞. Pulv. Cinchon. Cordifol. ʒss.

——Zingib. gr. iij. Mf. Pulv.

"On the eleventh day I saw her again; the medicines had been taken regularly, and she had passed, upon the whole, a better night." 50.

The pain gradually subsided, and in three or four days more it was removed. In cases, however, where the disease has been of long duration, much more time will be required before the salutary effects of the bark are perceptible. "But it must be persevered in, and taken regularly through the day and night, or it will not be found often to succeed." Here Mr. Swan states a case where the disease was brought on by a blow on the face, which broke one of the incisor teeth. In this instance two scruples of the bark, with an infusion of the same and cloves, were exhibited every three hours. These remedies removed the disease in a short time. A subsequent relapse was also cured by the same means.

The next case brought forward by Mr. Swan is to shew how debility produces a morbid sensibility of the nervous system, in general, and thus disposes to local complaints of particular nerves. The patient had eaten some hashed hare, which had stood in a brass pan covered with verdigrease, in consequence of which his health was much impaired. When Mr. Swan saw him, some months after the accident, he had an affection of the nerves at the back of the head, that caused excruciating pain. He had become weak and much emaciated, and had used a variety of means without any abatement of the complaint. Mr. S. ordered him half a drachm of the powdered cinchona every three hours, and a blister to the back of the neck. As the paroxysm was always most severe in the mornings, a draught with 30 drops of laudanum was ordered at that time, while wine and malt liquors were recommended for drink. Soon after this plan was adopted the pain began to be less severe, and it gradually diminished till it ceased entirely. As the pain diminished, his bodily health improved.

When the complaint has resisted bark, and the various other methods that have been recommended, as arsenic, belladonna, steel, mercury, &c. then our author considers the division of the nerve as our last hope, though too often a fallacious one. "Nonne verisimile sit," says Dr. Kerrison, "signis aliorum morborum perspectis, quod dolor hic originem ducat a quavis corporis parte longè distante, propter nervorum consensum." *Thesis, p.* 8. Dr. K. illustrates this by adverting to the irregular action of the voluntary muscles in chorea, where the cause of the disease is often unequivocally seated in the abdomen—to the pruritus narium from ascarides in the rectum, and also in the infantile remittent fever, where children will frequently continue to pick the nose and cheeks while any sordes remain in the bowels—the facial irritation immediately subsiding when the cause is removed by cathartics. Dr. Kerrison here asks if it be unlikely that depraved gastric or intestinal secretions should excite painful affections of the fifth pair of nerves from their consent, or rather connexion with the great visceral nerves. In the next sentence the Doctor asks what is this connexion? This question is answered by himself in a subsequent part of his Thesis. "The connexion of the second branch of the fifth pair with the great sympathetic, by means of the spheno-palatine or vidian nerve, is capable of being demonstrated." Hence, says he, we see the reason why dividing the facial nerves, where tic douloureux is sympathetic of visceral irritation, fails to procure relief, since the vidian junction of the fifth and great sympathetic, "profundior est quam scalpellus chirurgi peritissimi pertingere queat." 37. Dr. Kerrison seems persuaded that tic douloureux is not a local disease—but depending on a constitutional origin—"sedantium medicamentorum fere omnium applicatio jam dicta, atque multimodis repetita, æque ac nervorum sectiones *quas videram*, absque fructu quam minimo, mihi persuaserunt *neuralgiam facialem spasmodicam a causa generali pendere.*" Dr. K. from attentive observation and experience, has been led to conclude, that depraved secretions in the primæ viæ are the grand causes of this disease, and that although purgation may prove an auxiliary, it will not cure tic douloureux. His principal reliance is on the cinchona judiciously administered, with attention to regimen, open bowels, and other juvantia. He is unable to account for the ratio agendi of this valuable medicine; otherwise than that it changes or corrects the habit of mal-secretion in the stomach and other chylopoietic viscera.

"Absentia doloris capitis, remissio frequens, ademptio etiam totalis cruciatûs, absque remediis topicis, et non rarò sine opio aut quovis pharmaco narcotico, probant, ni fallor, *neuralgiam facialem spasmodicam nec a cerebro, cerebello, neque a nervo faciali quocunque originem trahere*; ergo, probabile ratus sum, ut cinchona, vi quâdam astringente prædita, superficieique purgatæ intestinorum admota, aliquid mutationis efficiat, quo facto, munus eorum magis salubriter perficiatur, ita ut liquor, gasve excitans, haud amplius secernatur, aut conficiatur, et *Irritatio Faciei symptomatica quiescat.*" *Thesis*, 44.

Dr. Kerrison states that he has used the following means in tic douloureux with very little effect, viz. repeated leeching of the part, cupping,* applications of ice, pouring cold water from a height, extract of belladonna externally, opium, plumbi carbonas, in form of ointment with oil, superacetas plumbi, fomentations of tobacco infusion, blisters, issues, (with some relief) electricity, prussic acid. In respect to the treatment, Dr. K. lays down the following therapeutical indications, viz. 1st, to cleanse the intestinal canal; 2d, to ameliorate the intestinal secretions. He properly considers it superfluous to dwell upon forms or prescriptions, since every one acquainted with physiology, pathology, and thera-

* General bleeding he has never tried, but in a note it appears that Dr. Pemberton had recourse to this measure in his own person, every second day till he could scarcely walk, without making the least impression on the complaint!

peutics, must be capable of combining and adapting remedies according to age and constitution. In order to fulfil the first indication, Dr. K. recommends from three to five grains of calomel to be taken at bed time; and the next morning some jalap and cream of tartar, or castor oil, or senna, varying the pill according to circumstances, but exhibiting it every third night, unless the gums should get tender, when it is to be omitted. With the mercurial preparation, our author sometimes combines antimony, and even recommends the exciting vomiting in some cases, as salutary.

To fulfil the second indication he depends, as we said before, on the cinchona, and prefers the following formula:—

℞. Extracti cinchonæ ℈j. ad ʒj.
Decoct. ——— fl. ℥xiv.
Tincturæ ——— ʒj. ad ʒiss. fiat haustus tertia vel quartaquaque hora sumendus. But as the patient is apt to get tired of so much bitter medicine, Dr. K. has occasionally given from two scruples to a drachm and a half of the extract of cinchona three or four times a day in wafer paper, washing it down with some water and tincture of bark or nutmeg water.

As a palliative, Dr. K. depends principally on opium, given in doses proportioned to the urgency of the case. In correspondence with his opinions, relative to the cause of the disease, he lays considerable stress on diet. He directs the breakfast to be made of tea, coffee, or chocolate, containing one-third part of milk, with bread and butter, and cold toasted bread. For dinner he thinks the patient should take some light soup with bread, followed by fish, or animal food in moderate quantity; but avoiding all vegetables, excepting good potatoes or turnips. For drink, water or weak brandy and water, is to be allowed. The patient is to use warm cloathing, and avoid the night air, wet, cold, and sudden atmospherical vicissitudes.

But to return to Mr. Swan. After giving directions for the division of different branches of the trigemini, he remarks of the portio dura of the auditory nerve, that —

"To attempt to divide the trunk of this nerve will not only be very difficult, but it will be likewise very dangerous: and to divide all the branches that go to the face, requires an incision from the zygoma to the angle of the jaw; but the greatest portion may be divided by making an incision down to the jaw, a little below the zygoma, and thus the main branches of the nerve will be cut through; and if the patient is not relieved by this operation, another incision may be made quite on the angle of the jaw, by which nearly all the principal branches will be divided." 56.

When the *third* branch of the fifth is affected, and the pain is in the side of the tongue and the teeth of the lower jaw, it would be hazardous to attempt its division. But when the lower lip is suffering from an affection of the nerve as it passes out of the foramen in the lower jaw, an operation may, of course, be performed by passing the point of a knife at the root of the first bicuspis tooth, between the lip and bone, down to the foramen, and moving it a little from side to side.

Before taking leave of tic douloureux we may here say a few words respecting the third work at the head of this article. The first lines of Dr. Villers' pamphlet engendered ominous anticipations in our minds. *In not one single case of tic douloureux has this author failed!* No wonder that, encouraged by such unprecedented success, he should come before the public—"regardless equally of the critic's acumen, or the irony of invidious competition, to give publicity to a mode of successful treatment in a disease which, it would appear, has hitherto set medical skill at defiance." P. 1. This *new* mode of treatment consists, besides a few common dietetic rules, in the following prescriptions:—

Formula 1. Ol. succini ℥j. gum. camph. ℈j. fiat embrocatio.
Formula 2. Ex. stramon. gr. ss. ex. col. comp. gr. v. ft. pil. om. nocte sum.
Formula 3. Pulv. cinchon.
Ferri carb. āā ʒss.
Mist. camph. ℥ij. m. ft. haustus sexta quaque hora s.
Formula 4. Extr. belladonn. gr. j. ex. stramon. gr. ss. hora s.s.
Formula 5. Ung. hyd. fort. ℥ij. g. camph. ℈j. ft. unguentum omni nocte utend. ʒss. in partem affectam.

Considering that all the above remedies have been tried, and have failed, over and over again, it is pretty well for Dr. Villers to say in page 8, that the various remedies in the hands of others "have been in vain resorted to, till the unfortunate patient is, in fact, *tortured out of his existence.*" We do not deem it necessary to go farther into this pamphlet. We would recommend the author, before he again throws the gauntlet to critics, to correct the orthography of tic doulòureux in the title page and various other places. He had better, too, revise some of the synonims, for instance, "dolor faciei nervorum *cruciandus.*" "Although," says he, "pre-eminence advances the sciences, yet the field of hitherto unexplored knowledge remains open, inviting the *speculist* (query, a mirrour maker?) to enter the lists of fame, and cull a twig from the luxuriant *branches* of the fertile *soil.*" P. 6. We have heard of branches of trees, rivers, candlesticks—and bible societies, but never before understood that literary laurels were to be plucked from branches of *soil.* We just throw out these friendly hints to the author, to convince him that few works are so perfect as to render it prudent for the writer to challenge criticism. For our own parts, we are not inclined to accept these literary challenges. We open books for the sake of gleaning information from them, and not to indulge in pugnacious and censorious disputations.

But to return again to Mr. Swan. In the 5th chapter, Mr. Swan takes up the subject of painful affections of other nerves than those of the face. He relates a case at page 58, where the neuralgic affection extended in the course of the ulnar nerve, from the elbow to the little and ring fingers, the pain coming on in fits, with disturbance of the digestive organs, and palpitations of the heart.

> " She used a spirituous embrocation for the arm, and took five grains of the mercurial pill at bed-time, and a mixture with camphor and the volatile tincture of valerian, by which the pain was diminished. She was then attacked by a severe affection of the uterus; and after some time, when she was recovering from this complaint, the pain in the nerve ceased entirely, and never returned." 59.

Mr. Earle has related a similar case in the 7th vol. of the Medico-Chirurgical Transactions, where the complaint was cured by cutting out a portion of the nerve. Mr. Abernethy has also published an interesting case of a young lady, where the complaint was seated in the integuments of one of the fingers, and which was ultimately cured by cutting out half an inch of the digital nerve. Mr. Swan therefore prefers the removal of a piece of nerve to a simple division of it, from which two advantages are derived—in the first place, " because the divided portions would be longer in uniting, and more time would therefore be afforded for the diseased action of the parts to wear off—and secondly, because they would retract out of the reach of the external wound, and be less liable to partake of any inflammation or other irritation occasioned by it."

From experiments made by Dr. Haighton and Sir Everard Home, it would appear that the uniting medium of nerves has the power of transmitting some nervous influence in the course of a few days, or even in twenty-four hours. Our author thinks it questionable whether the nerves have the power of communicating their influence to other nerves, whose communications with the brain have been cut off, in the same manner as the arteries have, whose direct communication with the main trunk has been intercepted by a ligature. At all events it can only exist in a very trifling degree. In a case related by Mr. Earle, where he cut out a portion of the ulnar nerve behind the inner condyle of the os humeri, the little finger remained nearly useless five years after the operation, and sensation was very imperfect.

VI. The 6th chapter is on inflammation of nerves. When a nerve is inflamed, (which generally takes place from contiguity to an inflamed part,) it becomes increased in size from a deposit of coagulable lymph between its fibres. Acute *idiopathic* inflammation of a nerve Mr. Swan believes to be of rare occurrence. Chronic phlogosis, however, is more common. It sometimes affects the extremities of nerves in amputated stumps. They appear enlarged in these cases, for some distance, and their extremities are swollen into a gangliform tumour. When the nerves are in this state the patient suffers so much pain from the least touch, as to be obliged to submit to a second amputation. In most cases of what are termed sciatica, Mr. S. believes the disease to be an inflammation of the neurilema frequently ending in an effusion of serous fluid.

"When people of a very robust habit are affected with this complaint, it is necessary to take some blood from the arm, at the same time local evacuations of blood are necessary; afterwards a large blister should be applied over the seat of the pain, and purgative medicines should be administered.

"When the complaint does not give way to these remedies, the extract of stramonium should be given in doses of a quarter of a grain, gradually increased to two grains, three times a day. In some instances, when every thing has failed, an entire confinement to bed, and tonic medicines, with as much opium or other anodyne as will moderate the pain, has been found to succeed.

"In obstinate cases, it will also be of use that an issue should be made near the trochanter, and that a grain of submuriate of mercury should also be given every night." 68.

To the above means we would recommend from forty to sixty minims of the *vinum seminis colchici* for a few nights at bed time, after large local evacuations of blood by means of leeches. We have recently had considerable success by these means in two or three cases of obstinate sciatica.

VII. *Ulceration of Nerves.* The following case in illustration of the subject we have considerably abridged. Wm. Sharpe, a soldier, ætat. 54, was wounded, at the age of 15, in the tibia, by a splinter of wood, followed by an exfoliation of bone, and healing of the wound, which continued well till the year 1814, when he received the kick of a horse, and the wound opened out again to an extent of six inches by three. Some exfoliations came away, and in 1817 it began to have a fungous appearance, with considerable hæmorrhages on being touched. Escharotics reduced the fungus to the level of the surrounding parts; but before this reduction he had suffered such violent pain in the thigh and leg, that he desired amputation, which at length was performed in June 1819, followed by perfect recovery in a month. On examining the limb, the bone was found enlarged upwards, with a considerable hollow in it opposite to the ulcer, occupied by the fungus. In this hollow the bone was spongy and soft, but none of its exterior part dead. The fungus seemed to grow from the hollow of the bone. The muscles of the leg had lost their natural character, and very much resembled fat. The sciatic nerve was much enlarged, and many of its branches proportionally so.

"The greatest part of the nerves was covered with a layer of a substance resembling fat, but very different in appearance from the other fat of the limb, or from that which is usually about the nerves; when freed from this, most of them had the nervous appearance, though they were apparently of a closer texture than sound ones. Several varicose veins were observed in different parts of the sciatic nerve. Some of the nerves were unusually soft, being easily torn. The following is a particular description of each nerve.

"The branch of the anterior crural nerve that accompanies the saphena major vein was somewhat enlarged; about an inch and a half above the ulcer it was still larger, and even with the ulcer, by which it was nearly surrounded; at the upper part it was firmly united to the adjacent parts for an inch and a half, after that it was loosely connected with them, and then again for rather more than two inches it was firmly united to them; near the malleolus internus it became of its natural size and appearance." 73.

Nearly the same may be said of the fibular and anterior tibial nerves. About an inch of the former nerve, however, was in a state of ulceration; "so much so, in one place, as to be nearly divided." The anterior tibial nerve enlarged proceeded firmly united all the way to the surrounding parts, and at one place, between the tibia and fibula, it was so blended and confounded with the textures in its vicinity, as hardly to be recognized. In this case our author concludes that the pain was owing to the dilated veins, as the patient never had any pain after the limb was amputated and the stump healed well—a clear proof, he thinks, that though the nerve was enlarged, it was not diseased; the enlargement and pain proceeding from the ulcerated state of the parts in the leg. Here Mr. Swan introduces a passage from Morgagni, to shew how violent the pain is when the nerves are in a state of ulceration. The case was an aneurism in the right groin, which extended backwards so as to produce ulceration in the sciatic nerve.

" 'In the last month the pains became most severe, not only in the tumour, but sometimes beneath the internal malleolus; in which place only, violent as the pains were, the foot was sensible, being in every other part deprived of feeling and motion. In all this month he neither had an interval of ease nor any sleep until his strength failed; then for some days he lay half asleep, and so died.

" 'On examining the limb, the nerve was so much eroded, that a few fibres hardly remained by which the superior and inferior parts were joined together.' " 76.

VIII. *Tumours in the Nerves.* These may be distinguished by the excessive pain which pressure occasions, and from its shooting generally in the exact course of the nerve, though Mr. Charles Bell, in his operative surgery, relates an exception to this rule, where a tumour in the ham did not, when pressed on, cause any particular pain, but rather a sense of pricking numbness down the leg. On dissection the sciatic nerve was found to enter into the substance of the tumour. Mr. Swan thinks it better to cut out the portion of nerve in which the tumour is seated, than to dissect the tumour out of the nerve. Here Mr. Swan instances cases from his own and the practice of others, illustrative of the foregoing observations.

IX. & X. *Divided Nerves.* If a nerve have been divided, and the external wound is healed by the first intention, very little pain is felt in the nerve, and sensation gradually, though slowly, returns. An open ulcer, however, connected with a wounded nerve, is generally very painful, and sometimes produces violent symptoms; consequently it is a matter of the greatest consequence, when a nerve is divided, to effect union of the parts around it by the first intention.

" When a nerve has been divided, and the external wound has healed, and there are marks of inflammation about the cicatrix, as a slight redness, tumefaction, and tenderness on pressnre, it frequently happens that this inflammation is communicated to the nerve and causes great pain, which is generally aggravated by any motion of the part.

" The best method of treatment will be to apply leeches near the parts, and evaporating lotions, and to keep them constantly at rest.

" I need not say how necessary it is in all diseases to pay proper attention to the state of the digestive organs, and that it is so most especially in all diseases and injuries of the nerves." 98.

In the following case the sciatic nerve was wounded by a fracture of the thigh bone—an accident, Mr. Swan thinks, of not very unfrequent occurrence.

"John Wright, about seventy years of age, got a fall about the beginning of May, and injured the left hip: I saw him for the first time on the first of June. The knee and foot were turned completely inwards; and if this position of the limb was changed, it was always soon resumed: the thigh could be raised by an assistant towards the abdomen as high as usual, but could not be rotated much: the limb was shortened about an inch: the trochanter major was not far from its usual situation, but behind it there was a rounded tumour, which was apparent, and could be distinctly felt, so as to convey the exact resemblance of the head of the thigh bone: when the hand was placed about the trochanter, and the limb was moved, a crepitus could be felt: the limb had the exact appearance of the dislocation backwards. He complained of very violent pain for some time, much more than is usual; but for the last two or three weeks he lay in an almost insensible state: he was in a very debilitated state before the accident, but after it he never had any appetite; so that he sunk from complete exhaustion on the twenty-fourth of June.

"The next morning I examined the part where the injury was received.

"On dividing the integuments, a small quantity of a dark-coloured fluid escaped: all the parts for some distance appeared one confused mass, from the quantity of coagulated extravasated blood. The thigh bone was broken through below the capsular ligament, and another portion was broken off below this, in an oblique direction, so as to leave the trochanter major nearly perfect; this portion lay behind the trochanter, and when covered by the integuments had a rounded feel like the head of the bone: another small portion was likewise completely detached. All the portions of bone were surrounded by coagulated blood, which appeared to have become organized, for in several parts of it were found osseous deposits: the head of the bone appeared inflamed, and was coated with coagulable lymph, Nearly all the cartilage lining the acetabulum was absorbed.

"The sciatic nerve was much enlarged, and likewise surrounded by coagulated blood; and in one place a portion of coagulum, about the size of a filbert, adhered very firmly to it; and when it was examined, portions of a whitish substance might very distinctly be seen in it, so as to convey the idea of this part having taken on the structure of newly-formed nerve: at this part some nervous fibrils had been lacerated." 103.

The appearances of the limb in this case differed from what are usually presented in fractures of the neck of the thigh bone. They might, without great care, have been mistaken for dislocation of the bone backwards. Under such a mistake how greatly would the patient's sufferings have been aggravated by extension of the limb!

XI. The eleventh chapter of Mr. Swan's work is on punctures or partial divisions of nerves. This accident may be suspected when very acute pain accompanies the infliction of a wound, especially if the pain extends in the course of the nerve, accompanied by convulsions or other symptoms of great nervous irritation. We shall abridge an interesting case of punctured nerve, communicated to Mr. Swan by Dr. Wilson of Grantham. Dr. W. was called to a woman labouring under strong convulsions. She had been bled two days before by a gardener, considerable pain being experienced at the time, shooting from the wound up to the shoulder. The wound was somewhat inflamed, and a thin liquor oozed from its lips. While making the examination, the woman became strongly convulsed. With the view of interrupting the communication from the diseased point to the sensorium, a tourniquet was applied above the part. A remission of the spasms soon followed, and an anodyne was administered; but the convulsions, after a short interval of ease, recurred as before, and another application of the tourniquet produced no good effect.

"As I had no doubt," says Dr. Wilson, "that the cause of the disorder was an injury of a cutaneous nerve in the operation of venesection, I determined to endeavour, by a transverse incision, to divide the nerve above the injured part, and to destroy its connexion with the sensorium; I therefore made an incision while the convulsions were most violent, of about an inch in length and small depth just above the orifice: no mitigation of symptoms was perceived; but on making another incision above the former one, somewhat deeper and longer, she cried out immediately, to the astonishment of the attendants, 'I am well, I am quite well, I can stir my arm;' which she began to move, and continued to do so with great delight for some time in various ways. She had no return of the spasms, and very soon got well." 109.

When a nerve has been wholly divided, each portion immediately retracts to some distance. When partially divided, the divided portions retract in the same manner, though not in the same degree. Each nerve being composed of different fasciculi, and these fasciculi generally communicating together, it follows that if a fasciculus be partially divided, or wholly divided where it communicates with its neighbour, an unnatural distention of parts will take place, accompanied with considerable pain. This will be still more the case if a nerve be wholly divided, with the exception of one fasciculus, which one will be greatly on the stretch. Still, as nerves must be totally or partially divided in almost all the accidents and operations to which the human frame is liable, it is difficult to account for the comparative rarity of any serious accidents succeeding these lesions. That the wound of a nerve may be the entire and immediate cause of the symptoms, independent of inflammation, or any thing else that could irritate the nerve, the following case, from Sabatier's Medecine Operatoire, is brought forward by Mr. Swan to prove.

" 'This slight operation,' he says, 'was very painful, and was soon followed by convulsive motions, which extended themselves through the whole of the wounded extremity, and then through the rest of the body: these symptoms were not accompanied by any tumefaction, and were very often renewed. The patient could neither walk, nor ride in a carriage. This state having continued a long time, notwithstanding the use of antispasmodics and quieting remedies, I advised a division of the saphenus nerve, but it was not consented to; nevertheless the nervous symptoms gradually diminished, and the patient partly recovered her health, after five or six years almost continual suffering.' " 114.

Mr. Swan would advise, in cases of injured nerve from bleeding, that several leeches be applied to the neighbourhood of the part, and afterwards evaporating lotions. Should these disagree, poppy fomentations and poultices are to be tried, keeping the limb as quiet and easy as possible. He has no doubt that the greater number of injured nerves in venesection are rendered troublesome by too much exertion of the arm inducing inflammation. We have had reason to believe, that the state of the atmosphere has sometimes an effect in festering venesection wounds—at least during the severe winter of 1812-13, in Holland, a great number of men who were bled for pneumonia, and who used no muscular efforts at all afterwards, had the wounds inflamed, and that without any suspicion of bad lancets or improper modes of opening the veins. Mr. Swan relates the following case to shew that if a nerve be wounded in bleeding, and the external wound heals by the first intention, the wound of the nerve will not be of much consequence.

" I bled Mrs. D. in the median cephalic vein; she complained of very acute pain at the time I made the puncture, and it continued for several hours.

" As I was certain from the manner in which she complained that I had wounded a nerve, I was very careful in binding up the arm well, so as to keep the lips of the wound in exact contact, and at the same time told her of the necessity there was for keeping her arm entirely at rest. The wound healed by the first intention, and the pain did not return." 116.

Mr. Swan is of opinion that in whatever way nerves are wounded, the lesions are repaired by Nature alone. It is therefore right, when an accident of this kind happens in venesection, to wait for a while and try palliatives. But if the irritation induce convulsions, as in the case related by Dr. Wilson, and threaten life, an operation should be attempted in order to cut off the communication between the brain and the wounded nerve. Mr. Swan quotes two cases from M. Larrey, in elucidation of this principle, although the accidents did not arise from venesection.

"The first was that of a man who was struck by a ball, which crossed the right arm, and wounded the biceps and coraco-brachialis muscles, and the radial and internal cutaneous nerves. On the eighth day he began to have great pain; and it was wished to divide a bridge left by the wound, in which were found some branches of the internal cutaneous nerve, but the patient refused to have it done. The next day his local pains were very acute; he had convulsive motions of the hand and fore arm, heat in the whole system, and locked jaw; he was very restless, and in continual agitation. The rapid progress of the symptoms determined Larrey to divide the bridge, and cut the bottom of the wound, where he found several nervous bridles. This operation was very painful; but two hours afterwards the patient was much relieved, and in the space of two days all the symptoms disappeared.

"The second was that of a man who received an injury from a spear on the right side of the forehead. The point of the spear had slided obliquely from below upwards and inwards under the pericranium, so as to make a deep fissure in the frontal bone: one of the superciliary nerves was grazed by the cutting side of the spear.

"Nine days passed without any alarming symptoms, and it was considered as a simple wound; but in the night between the ninth and tenth days tetanus came on, with convulsive motions of the corresponding eye-lids, and a loss of sight in that eye: there was a little mental wandering, a very acute pain, locked jaw, and a very marked disposition to emprosthotonos.

"Emollients were immediately applied to the wound, and diaphoretic and opiate draughts were given without effect; the complaints went on increasing, and in twenty-four hours would have been at their greatest height. The wound was then examined with a probe, which gave very acute pain; this determined Larrey to divide from below upwards with a bistoury the whole of the superciliary muscle, the nerves, and vessels: the patient was immediately relieved, and in less than twenty-four hours all the tetanic symptoms had disappeared." 120.

Mr. Swan next extracts a case from Sabatier, which he thinks may throw some additional light on the subject of wounded nerves. A young man received a stab near the knee, at the inferior and inner part of the left thigh, in the course of the saphena vein and nerve. After considerable hæmorrhage, tumefaction and fever supervened, the affected extremity being very painful. When these symptoms had abated, a trembling and nervous agitation of the leg and thigh came on, and resisted all attempts at alleviation. M. Sabatier thrust a sword through the thigh of a dead subject, in the same direction, and found the saphena entirely divided, and the nerve cut half through. He therefore advised the cautery, to which the young man would not submit. The patient continued lame for some months, but, ultimately, though slowly, recovered. We must pass over Mr. Swan's remarks on tetanus, as nothing particular occurs in that portion of the work.

XII. & XIII. The twelfth chapter is on ligature of nerves, and the thirteenth on compression of them. The former is short, and does not contain any thing novel. The thirteenth chapter contains several interesting cases, but we find our limits overstepped, and shall only be able to make one extract as an example. The case is introduced by Mr. Swan to shew that when there has been a paraplegia, it is necessary to draw off the urine, not only on account of the bladder, but because the over-distension of this organ causes pressure on the nerves, and thus tends to prevent their recovery.

" Nov. 6, 1819. Mr F. aged twenty-five, had the heart-burn, after which he ate a hearty dinner of beef and apple dumpling, and the heart-burn went off. Soon after he carried a weight of wood for some distance, which produced the heart-burn again, and he was obliged to lie down on the damp ground from feeling so unwell. After he had laid a little while his legs began to feel weak, and he had much difficulty in walking home, as the powers of the muscles kept diminishing, and at last they entirely failed. The bladder, and also the sphincter ani, lost almost entirely their muscular power: the paralysis extended from the pit of the stomach downwards: he had no power of moving any of the muscles: he felt well, except being thirsty, and had a good appetite: when the bladder was very much distended he felt pain, but it was not violent: the feeling of the skin was so far perfect as to render him sensible of the slightest touch; but moderately cold things applied to the skin did not feel cold, nor did hot things feel more than just warm: his sensations of heat and cold with the upper extremities were perfect, and very different from those experienced by the lower.

" The symptoms in this case are slighter than those usually met with where pressure is made on the medulla spinalis, as in fractures of the spine; and, I think, prove, beyond all doubt, that the nerves have all the same degree of feeling, but that the various agents that call them into action act in a more easy or difficult manner. The sense of touch is by far the most mechanical; moderate degrees of heat and cold are less active agents; and the will still less.

XIV. Mr. Swan's fourteenth chapter contains numerous experiments on animals for the purpose of ascertaining the process which Nature employs for repairing wounds of nerves. Into these experiments we cannot enter, as they require to be read with care, and cannot be abridged. From the concluding chapter we shall make some extracts.

"It will be seen from these experiments, in the first place, that after a division of a nerve, the extremities of the divided portions become enlarged and more vascular, but especially the upper portion; and coagulable lymph, having the appearance of white of egg, is effused, which soon becomes vascular. In a few days the coagulable lymph from each portion becomes united, and anastomoses from between the blood-vessels; the coagulable lymph gradually assumes a firmer texture, and the number of the blood-vessels diminishes, and the newly-formed substance appears to contract, like all other cicatrices, so as to bring the extremities of the divided portions nearer and nearer to each other. It is difficult to determine from an experiment on the limb of an animal the exact time at which the nerve again performs its functions. In eight weeks after the division of the sciatic nerve, I have observed a rabbit to be in some degree improved in the use of its leg, but at the end of eighteen weeks it was not perfect. When the nerves of the leg of a horse have been divided just above the foot, they are sufficiently restored to perform their functions in a very great degree in six or eight weeks; but it must be observed that these nerves are only formed for sensation, and it is very different with the nerves of voluntary motion." 184.

Though this is the general way in which a divided nerve reunites, yet the reunion is sometimes accomplished by granulations. It appears also from Mr. Swan's experiments, that when a portion of nerve is cut away, the same restorative process is set up as when there is merely a division of the nerve; but the divided portions appear never to be again restored to the same size as the original nerve. The following curious circumstance we shall here quote.

"A horse had been lame for two years, at the end of which time an inch of each nerve going to the foot was cut out; after this he went very well for six months, when he again became lame, and continued so five months; at the end of this time he appeared to suffer such dreadful pain that he was killed. At the time he was operated on, it was supposed that the disease was the same in both fore legs, so that portions of the nerves of both of them were removed.

"On examining the legs after he was killed, one was very much swelled especially at the foot, where matter was discharged by several sinuses leading to the coffin bone, which was quite carious.

"On further examination, the nerves of this leg were found to have reunited, and the new-formed substance was very large, and appeared to have the same structure as that which forms the bond of union when a nerve has been simply divided. The nerves, above the place where they were divided, were found to be much larger than those of the opposite leg in the same place. In the opposite leg, in which there did not appear to be much disease, the nerves had reunited, but the bond of union was not so large as in the other leg.

"From these circumstances, it appears to me that the functions of the nerves were again performed, through the medium of the new-formed substance; but I am informed this is not usually the case when so large a portion as an inch of the nerve has been removed; and this circumstance shows, that in this instance it must have been owing to the irritation occasioned by the disease in the foot." 186.

Another conclusion deduced from our author's experiments is, that when a nerve has been included in a ligature, the parts to which it is distributed are deprived of sensation and motion, in the same manner as when the nerve has been divided. Immediately after the application of the ligature, the vessels of the nerve begin to enlarge and become numerous, coagulable lymph being, in the mean time, effused from the nerve above and below the ligature, through which the vessels shoot and anastomose. The ligature becomes incased by the lymph. Immediately after it is cast off, the separated portions of nerve begin to unite, and the process of reparation goes on until the union is so complete as to enable it to perform its functions.

But we must conclude. We hope we have done justice to Mr. Swan's work, which we conceive to be highly creditable to his zeal and talents. We trust that he will persevere in the investigation of abstruse points (of which there are, unfortunately, too many) in medical science, and for which investigation Mr. Swan's abilities appear to be well calculated. The plates of this work are very well executed.

